# The Independent Role of Body Mass Index (BMI) and Severity of Depressive Symptoms on Biological Changes of Women Affected by Overweight/Obesity

**DOI:** 10.3390/ijerph18062923

**Published:** 2021-03-12

**Authors:** Simona Iodice, Alessandro Ceresa, Cecilia Maria Esposito, Francesco Mucci, Diana Misaela Conti, Laura Pergoli, Letizia Tarantini, Luisella Vigna, Valentina Bollati, Massimiliano Buoli, Marta Serati, Stand-Up Project Group

**Affiliations:** 1 EPIGET LAB, Department of Clinical Sciences and Community Health, University of Milan, Via San Barnaba 8, 20122 Milan, Italy; simona.iodice@unimi.it (S.I.); laura.pergoli@unimi.it (L.P.); letizia.tarantini@unimi.it (L.T.); valentina.bollati@unimi.it (V.B.); 2 Department of Pathophysiology and Transplantation, University of Milan, 20122 Milan, Italy; cecilia.esposito@unimi.it (C.M.E.); francesco.mucci@unimi.it (F.M.); massimiliano.buoli@unimi.it (M.B.); 3 Occupational Health Unit, Center of Obesity and Work EASO Collaborating Centers for Obesity Management, Fondazione Ca’ Granda Ospedale Maggiore Policlinico, 20122 Milan, Italy; diana.misaela@gmail.com (D.M.C.); luisella.vigna@policlinico.mi.it (L.V.); 4 Department of Neurosciences and Mental Health, Fondazione IRCSS Ca’ Granda Ospedale Maggiore Policlinico, Via Francesco Sforza 35, 20122 Milan, Italy; 5 Department of Mental Health, ASST Rhodense, 20024 Rho, Italy; martaserati@libero.it

**Keywords:** depression, inflammation, hormones, clock genes, interleukins, gender

## Abstract

*Background*: Both obesity and depression are medical conditions associated with severe disability and biological abnormalities. Our aim was to study associations between Body Mass Index (BMI), depression and biological changes in women affected by overweight or obesity. *Methods*: Depressive symptoms were evaluated by the Beck Depression Inventory II (BDI-II) questionnaire in 200 women affected by overweight/obesity (mean age of the sample 52.7 ± 12.9 years, BMI 33.8 ± 5.5 kg/m^2^). A blood sample was obtained for evaluation of biochemical (oxytocin and vitamin D), inflammatory and epigenetic (methylation of clock genes) parameters. Multivariable linear regression models were used to study the association between BMI or severity of depressive symptoms (BDI-II scores) with different biomarkers. *Results*: BMI was found to be associated with severity of depressive symptoms (*p* = 0.050). Severity of obesity resulted to be associated with lower plasma levels of oxytocin (*p* = 0.053), vitamin D deficiency (*p* = 0.006) and higher plasma levels of IFN-γ (*p* = 0.004), IL-6 (*p* = 0.013), IL-7 (*p* = 0.013), TNF-alpha (*p* = 0.036) and chemokine ligand 3 (CCL3) (*p* = 0.013, R^2^ = 0.03). Severity of depression was significantly associated with more methylation of clock genes CRY1 (*p* = 0.034, R^2^ = 0.16) and CRY2 (*p* = 0.019, R^2^ = 0.47). More severe depression together with higher levels of IL-8 strongly predicted lower methylation of CLOCK gene (*p* = 0.009); *Conclusions*: Different biological abnormalities have been found to be independently associated with BMI and severity of depressive symptoms in women affected by overweight/obesity. The complex interplay between overweight, depression and biological changes will have to be better clarified by future studies.

## 1. Introduction

Overweight and obesity represent a serious public health problem as these conditions are estimated to cause more than millions of deaths in one year, 3.9% of years of life lost and 3.8% of disability-adjusted life-years worldwide [1]. A robust literature has associated obesity to a number of other medical conditions including diabetes, cardiovascular disease, some forms of cancer and also mood disorders so much that some authors have coined the term “metabolic-mood syndrome” [2]. Of note, a high co-occurrence of obesity and depression has been also reported; subjects affected by obesity have an increased risk to develop depressive symptoms, especially in case of comorbidity with diabetes [3].

Obesity and depression share a number of biological abnormalities. Lower levels of oxytocin, a hormone involved in different human behaviours including social attachment, parent-infant bonding, interpersonal relationships, has been found to be associated with severity of depressive symptoms during the perinatal period [4,5]. Similarly, oxytocin appears to regulate human metabolism and play a role in weight regulation [6]; patients with obesity have reported to have mean lower oxytocin plasma levels than healthy controls particularly in case of comorbidity with diabetes [7]. Of note, intranasal administration of oxytocin in animals and humans with obesity has shown to be associated with improvement of excessive appetite and reduction of food intake [8]. Furthermore, different studies have also reported lower levels of vitamin D in patients affected by depressive disorders [9] as well as in subjects with obesity [10]. Over-inflammation has been also described for patients affected by mood disorders [11] or obesity [12]. This is not surprising, as cytokines of innate immunity such as interleukin 6 (IL-6) and tumor necrosis factor alpha (TNF-α) may be produced by adipocytes as a result of low-grade inflammation of white adipose tissue which characterizes obesity [13]. Furthermore, increased levels of IL-6 and TNF- α have been reported both in depressed patients and in those with obesity [11,12].

Finally, abnormalities of circadian rhythms and consequently of expression of genes related to “clock system” have been described for both patients affected by depression and subjects with obesity. Depression, in particular the melancholic classical type, is associated with a global advance of circadian rhythms [14]. Sleep rhythms are generally deeply disrupted in severe depressed patients, including women with perinatal depression [15] and melancholic subjects that tend to have early sleep and late insomnia [14]. Two recent reviews highlighted that, despite the contrasting results, polymorphisms of Cryptochrome Circadian Regulator 1 (CRY 1) (e.g., the single nucleotide polymorphism-SNP rs2287161) may be potentially associated with an increased risk of Major Depressive Disorder (MDD) [16]. Chrono-disruption has been extensively described also in obese patients as well as an altered methylation and consequent expression of circadian genes [17]. In addition, SNPs of clock genes particularly of Clock Circadian Regulator (CLOCK) and CRY1 genes have been detected as potentially associated with the development of obesity [18].

In this framework the objective of this study was to analyse the different contributions of Body Mass Index (BMI) and depression in the dysregulation of the biological mechanisms of women affected by overweight or obesity. Our question is if the greater clinical severity of depressed and overweight subjects may be the result of a more profound alteration of common biological mechanisms or if depression and BMI entail a dysregulation of different biological aspects. In the light of these considerations, the aim of this study is to find biological factors which may predict the clinical course of overweight patients, as further biological changes associated with depressive symptoms are expected to negatively impact on quality of life and social functioning of these women. The results of the present research are supposed to be useful to implement prevention strategies and to identify new targets for pharmacotherapy.

## 2. Materials and Methods

### 2.1. Participants

The study was conducted at the Department of Psychiatry in collaboration with the Epidemiology, Epigenetics and Toxicology Lab-Department and the Worker’s Health Protection and Promotion Unit, Fondazione IRCCS Policlinico, University of Milan. The study design, research aims and measurements have been approved by the local Institutional Review Board (‘Fondazione IRCCS Ca’ Granda Ospedale Maggiore Policlinico’ review board, approval number 1425).

The subjects were selected among women visited in the Occupational Medicine Centre of Ospedale Maggiore Policlinico. The eligibility criteria of the study were: (1) older than 18 years at enrollment; (2) affected by overweight/obesity according to BMI following definitions: overweight-BMI between 25 and 30 kg/cm^2^; obese-BMI of 30 kg/cm^2^ or more; (3) resident in the Lombardy Region at the time of recruitment. Exclusion criterion was pregnancy. We identified the 443 women who had been screened by the *Beck Depression Inventory II* (BDI-II) questionnaire at the time of blood sample for the biological analyses. BDI-II is considered a proper tool to evaluate depressive symptoms in women with medical comorbidities such as obesity [19]. The women with a BDI-II score ≥14 (N = 100) have been selected for the purpose of this study, as they are supposed to present clinically significant depressive symptoms [19].

These depressed women were matched with 100 of the 343 remaining non-depressed women (BDI-II score <14), using a 1:1 randomization method. The final population we tested consisted of 200 women, stratified according to BDI-II scores (BDI-II score <14-no depression: *n* = 100; BDI-II score 14–19-mild depression: *n* = 13; BDI-II score 20–29-moderate depression: *n* = 74; BDI-II score 30–63-severe depression: *n* = 13).

The following data (as well as BDI-II scores) have been considered:

- clinical variables: age, BMI, current use of antidepressant, Homeostatic Model Assessment for Insulin Resistance (HOMA) index;
- biochemical parameters: oxytocin plasma levels, vitamin D deficiency (plasma levels <20 ng/mL) adjusted for season, homocysteine, % neutrophils;
- inflammatory markers: granulocyte-macrophage colony-stimulating factor (GM-CSF: it favours the maturation of stem cells in leukocytes), interferon gamma (IFN-γ: pro-inflammatory cytokine which stimulates macrophage), interleukin 2 (IL-2: pro-inflammatory cytokine which stimulates T cells), interleukin 3 (IL-3: it favours the differentiation of stem cells in myeloid progenitor cells), interleukin 4 (IL-4: it favours differentiation of naïve t helper (Th) cells in Th2 ones), interleukin 6 (IL-6: multi-functional cytokine), interleukin 7 (IL-7: it favours the differentiation of stem cells in lymphoid progenitor cells), interleukin 8 (IL-8: it is a neutrophil chemotactic factor), interleukin 10 (IL-10: anti-inflammatory cytokine), interleukin 18 (IL-18: pro-inflammatory cytokine which induces cell-mediated immunity), chemokine ligand 2 (CCL2: it attracts monocytes at the site of inflammation), chemokine ligand 3 (CCL3: it attracts macrophages and neutrophils), tumor necrosis factor alpha (TNF-α: pro-inflammatory cytokine of innate immunity), tumor necrosis factor beta (TNF-β: pro-inflammatory cytokine of innate immunity);
- epigenetic markers-rate of methylation of the following clock genes: Brain and Muscle Aryl Hydrocarbon Receptor Nuclear Translocator-like Protein-1 (BMAL1), Circadian Locomotor Output Cycles Kaput (CLOCK), Cryptochrome Circadian Regulator 1 (CRY1), Cryptochrome Circadian Regulator 2 (CRY2), Period Circadian Regulator 1 (PER1), Period Circadian Regulator 2 (PER2), Period Circadian Regulator 3 (PER3).

### 2.2. Laboratory Methods

The plasma levels of oxytocin, vitamin D and the inflammatory parameters were measured through enzyme-linked immunosorbent assay (ELISA), while the methylation status of the clock genes in the leukocytes was investigated through the pyrosequencing process. Of note, DNA methylation of peripheral leukocytes is considered a good marker to make distinction between depressed versus non-depressed patients [20]. With regard to pyrosequencing, one µg DNA (concentration 50 ng/µL) has been bisulfite-treated using EZ DNA Methylation-Gold™ Kit according to the manufacturer’s protocol. Final elution has been performed with 30 µL of M-Elution Buffer. Bisulfite-treated DNA has been stored at −80 °C and used shortly after treatment. For each reaction, a 50 µL PCR has been carried out in 50 µL of GoTaq Green Master mix, 1 pmol of the forward primer, 1 pmol of the reverse primer, 50 ng of bisulfite-treated genomic DNA and water. One of the primers is biotin-labelled and it is used to purify the final PCR product by Sepharose beads. The PCR product has been bound to Streptavidin Sepharose HP and the Sepharose beads containing the immobilized PCR product purified, washed, denatured using a 0.2 M NaOH solution, and washed again using the Pyrosequencing Vacuum Prep Tool, as recommended by the manufacturer. Then, 0.3 µΜ pyrosequencing primer has been annealed to the purified single-stranded PCR product and pyrosequencing has been performed using the PyroMark MD Pyrosequencing System. Methylation quantification has been performed using the provided software. The degree of methylation has been expressed as % 5-methylated cytosines (%5mC) over the sum of methylated and unmethylated cytosines. We have used built-in controls to verify bisulfite conversion efficiency. Every sample has been tested three times for each marker to confirm reproducibility of our results.

### 2.3. Statistical Analyses

Sample size calculation was based on the association of BMI with severity of depressive symptoms. We estimated that 200 subjects achieve 90% power to detect a change in slope of from 0 under the null hypothesis to 0.23 under the alternative hypothesis when the standard deviation of the BMI is 5.5 kg/m^2^, the correlation is 0.24, and the two-sided significance level is 0.05.

Descriptive analyses of the clinical and outcome variables of the total sample have been performed. Regression assumptions were checked by performing diagnostic tests for each model, to verify normality of residuals and the homogeneity of the variance of residuals, for this reason methylation variables were log-transformed before applying linear regression models. Moreover, for each subject methylation measurements were done for different CpG dinucleotide positions replicated in two measurements. To consider intra-individual correlation due to repeated-measure data structure we constructed linear mixed models.

A first univariate linear regression model was performed to evaluate the association between overweight (BMI) and severity of depressive symptoms:

- BMI and oxytocin/vitamin D levels (corrected for Homeostasis Model Assessment–HOMA index);
- BMI and levels of inflammatory parameters (corrected for HOMA index);
- BMI and methylation status of clock genes (corrected for HOMA index, percentage of neutrophils, homocysteine plasma levels);
- Severity of depression (BDI-II scores) and oxytocin/vitamin D levels (corrected for HOMA index);
- Severity of depression (BDI-II scores) and levels of inflammatory parameters (corrected for HOMA index);
- Severity of depression (BDI-II scores) and methylation status of clock genes (corrected for HOMA index, percentage of neutrophils, homocysteine plasma levels, vitamin D deficiency). Of note, as well as neutrophil percentage, both HOMA index and homocysteine plasma levels have been found to influence methylation of genes in overweight subjects.

A further linear regression model has been performed to verify if the rate of inflammation could act as effect modifier of the association between severity of depression and clock gene methylation (e.g., as showed by higher plasma levels of IL-8).

A two-sided *p*-value < 0.05 was considered statistically significant. Statistical analyses were performed with SAS software (version 9.4, SAS Institute Inc., Cary, NC, USA). Finally, we calculated q-FDR values using the multiple comparison method based on Benjamini-Hochberg False Discovery Rate (FDR) that takes into account the high number of comparisons, with a threshold of 0.10 to detect significance.

A two-sided *p*-value < 0.05 was considered statistically significant. Statistical analyses were performed with SAS software version 9.4.

## 3. Results

The descriptive analyses of the clinical variables of the total sample are reported in Table 1. The sample consisted with 200 women with a mean age of 52.7 (±12.9) and a BMI of 33.8 (±5.5). Thirteen women (6.5%) presented severe depressive symptoms at the time of assessment.

**Figure 1:**
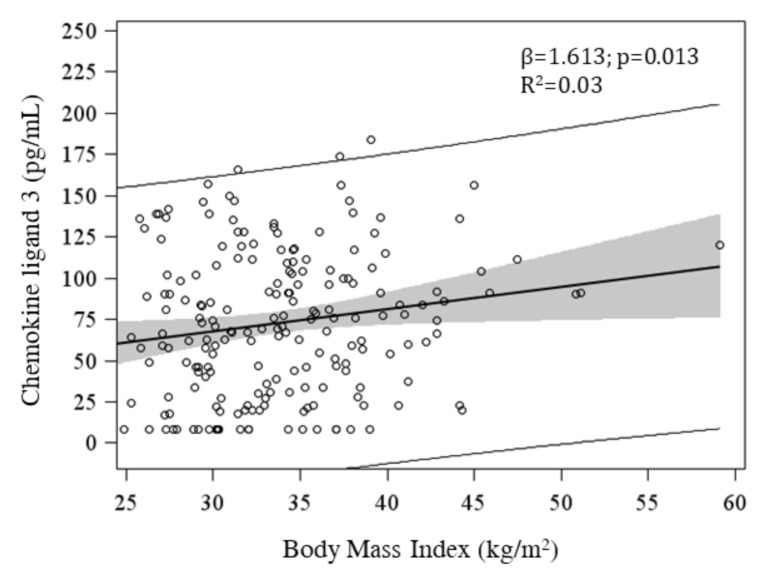
Association of BMI with plasma CCL3. Model was adjusted for HOMA-IR index. CCL: chemokine ligand; CI: Confidence Interval; GM_CSF: granulocyte-macrophage colony-stimulating factor; IL: interleukin; IFN-**γ**: Interferon gamma; q-FDR: False Discovery Rate; TNF-α: Tumor Necrosis Factor alpha; TNF-β: Tumor Necrosis Factor beta.

BMI has been found to be directly associated with severity of depressive symptoms, in the total original sample (N = 443) (β = 0.129, *p* = 0.05), with a trend towards statistical significance in the 200 selected women (β = 0.230, *p* = 0.093). Similarly, the severity of obesity resulted to be associated with lower plasma levels of oxytocin (β = −1.17, *p* = 0.053) and vitamin D deficiency (OR = 1.13 (IC 95%: 1.04–1.21), *p* = 0.002), adjusted for season. BMI has been found to be positively associated with a number of inflammatory parameters: IFN-γ (β = 0.235; *p* = 0.004), IL-6 (β = 0.043; *p* = 0.013), IL-7 (β = 0.164; *p* = 0.013), TNF-alpha (β = 0.148, *p* = 0.036) and CCL3 (β = 1.61, *p* = 0.013) (Table 2; Figure 1). Finally, methylation of none of clock genes resulted to be associated with BMI (Table 3).

**Figure 2:**
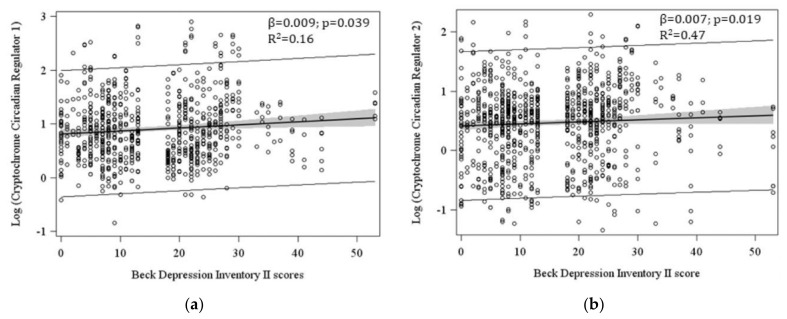
(**a**) The scatterplot reports CRY1 repeated 6 times for each subject as the methylation was measured at 3 CpG dinucleotide positions and 2 measurements; (**b**) The scatterplot reports CRY2 repeated 8 times for each subject as the methylation was measured at 4 CpG dinucleotide positions and 2 measurements. Models were adjusted for Homa-IR index, percentage of neutrophils, homocysteine plasma level, vitamin D deficiency, position of methylation, taking into account repeated measures.

**Figure 3:**
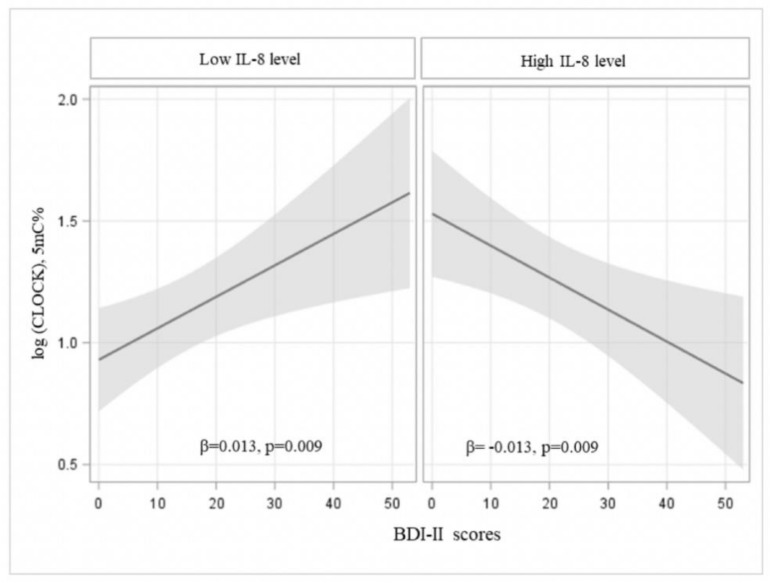
Association of BDI-II scores with methylation of CLOCK gene according to the changes of IL-8 plasma levels. Multivariable regression models applied to logarithmic transformation of clock gene, adjusted for Homa-IR index, percentage of neutrophils, homocysteine plasma level, position of methylation, vitamin D deficiency, IL-8, interaction between BDI-II score and IL-8. The estimates in the plot are calculated at mean level of continuous covariates and reference level of categorical variables (third position, no vitamin D deficiency). Low and high levels of IL-8 refers to 5° and 95° percentiles of its distribution (IL-8 low = 4.2 pg/mL, IL-8 high = 18 pg/mL).

Severity of depressive symptoms (BDI-II scores) has not be found to be associated with oxytocin plasma levels, frequency of vitamin D deficiency or inflammatory parameters. In contrast, severity of depressive symptoms was significantly associated with more methylation of clock genes CRY1 (β = 0.009, *p* = 0.034) (Figure 2) and CRY2 (β = 0.007, *p* = 0.019) (Figure 2). Furthermore, more severe depressive symptoms together with higher levels IL-8 strongly predicted lower methylation of CLOCK gene (β = 0.013, *p* = 0.009) (Figure 3).

## 4. Discussion

The first result of this study is that BMI seems to influence the severity of depression; women with more severe obesity are likely to have more severe depressive symptoms. Previous studies have shown that BMI is associated with a chronic course of MDD or re-hospitalization, although these negative effects may be mitigated by a collaborative care management of these patients [21]. An association between BMI and risk of depression has been also confirmed in large epidemiological studies in general population or in specific groups such as elderly subjects [22].

The results of this study show also that BMI plays a role in different biological changes. Women with more severe obesity have lower oxytocin plasma levels and a higher frequency of vitamin D deficiency. Other authors reported lower plasma oxytocin levels in subjects with obesity than healthy controls [7] as a result of the role of this hormone in reward-driven food intake [23] and energy metabolism [24]. Furthermore, it has been demonstrated that this hormone influences human social behavior and it may be protective against the onset of depressive symptoms especially in women and during the peripartum [25]. Similarly, the relation between vitamin D deficiency and BMI has been previously reported [26]. Of note, also vitamin D deficiency has been identified as a biomarker of increased risk of depression particularly during the perinatal period [9]. Overall, subjects affected by obesity would have biological abnormalities that would make them more susceptible to depression [27].

One of the proposed mechanisms that would link depression and overweight would be a dysregulation of inflammatory mechanisms since hormones like oxytocin [28] and vitamin D [29] would have an anti-inflammatory function. In support of this hypothesis, the results of the present study show a direct association between plasma levels of different inflammatory markers (IFN-γ, IL-6, IL-7, TNF-alpha, CCL3) and BMI. Several authors reported increased inflammatory markers in subjects with obesity than controls; interestingly one of this study reported increased plasma levels of the same cytokines of our sample, but in a population different from ethnicity and probably diet (African American women) [30], suggesting that perhaps some biological factors play a major role in obesity and are independent from environmental aspects such as type of diet [31]. In addition, preliminary evidence indicates that the pro-inflammatory interleukin 1 (IL-1) stimulates the release of CCL3 by human preadipocytes [32] and that gene expression of CCL3 and of its receptor is increased in adipose tissue of overweight patients [33]. Similarly, increased levels of CCL3 have been reported in depressed patients with respect to healthy controls [34]. In this sense, it is interesting to point out that physical exercise can induce molecular modifications which swerve a chronic pro-inflammatory state (which is present both in obesity and depression) to anti-inflammatory one [35]. So, physical training in obese women does not only cause a reduction of BMI with an amelioration of global health state [36,37], but it is also efficacious in treating mild to moderate depression [38], inducing increased levels of anti-inflammatory cytokines and of Peroxisome Proliferator-Activated Receptor-Gamma Coactivator Alpha (PGC1α), which reduces the synthesis of pro-inflammatory cytokines [35].

With regard to severity of depressive symptoms, our data support a dysregulation of the expression of clock genes and this result is not surprising as previous studies showed as specific polymorphisms of CRY1 make subjects more vulnerable to depression [16] and abnormalities of circadian rhythms (e.g., late insomnia) are typical features of patients with depression [15]. However, the most interesting result is perhaps the hypo-methylation of the CLOCK gene in depressed subjects with increased IL-8 levels. Knockout mice for the CLOCK gene showed a lengthening of the circadian rhythms [39] and this is consistent with our data showing a hyper-expression of the CLOCK gene and therefore a shortening of the circadian rhythms that is typical of the depressed subjects who tend to have an early sleep and a terminal insomnia. In addition, in agreement with our results, there are some reports about increased levels of IL-8 in depressed women [40] especially if suffering from somatic symptoms.

Surprisingly, in our sample none of the included biomarkers were both influenced by BMI and severity of mood symptoms although previous research studies found at least a partial overlap in biological changes associated with obesity and depression [41]. The apparent independency of BMI and depression in influencing biological parameters of our sample may have different explanations. One is that biological changes due to BMI and depression are truly independent and in this case the co-occurrence of depression and obesity would be responsible for more severe biological changes with respect to subjects with only one of the two conditions. Comparative studies with non-depressed subjects affected by obesity, depressed subjects with normal weight and depressed individuals with obesity could clarify this aspect. Alternatively, the biological changes due to obesity and depression are not simultaneous: depressed individuals could have a dysregulation of expression of clock genes and consequently disrupted circadian rhythms with the onset of obesity and over-inflammation. In this case prospective studies with depressed patients could better assess the risk of obesity in depressed individuals and the time of the associated biological abnormalities. Of note, not only obesity has been associated with depression, but also depressed patients would seem at risk of overweight [27]. A third explanation may be that biological changes due to depression may be masked by overweight because most studies about biological changes in depression have included normal weight subjects.

## 5. Conclusions

Globally, the results of this study highlight a complex interplay between depression, obesity and biomarkers. Both BMI and depressive symptoms resulted to be significantly associated to biological alterations in women affected by overweight or obesity. In particular, a higher BMI seems to induce more inflammatory and hormonal alterations, while more severe depressive symptoms may modify the expression of clock genes, although we did not find surprisingly any biological alteration shared by overweight and depression. This fact might have different explanations. First of all, BMI and depression may act independently each other in altering biological parameters of women affected by overweight or obesity. Another hypothesis is that obesity may hide biological alterations traditionally associated with depression [42]. Finally, another possible explanation is that biological changes connected to obesity and depression are not simultaneous: depressed women may develop an alteration of circadian rhythms which is an important risk factor for weight gain [43]. Obesity and depression are frequently in comorbidity, so that the study of the overlapping biological pathways underlying these conditions, both associated with adverse health outcomes, may allow to identify new pharmacological targets and to implement prevention strategies [44].

It has to be taken into account that the results may have been influenced by the specific features of this sample although the only inclusion of women has the advantage to avoid the bias of gender-related differences in some biomarkers such as hormones (oxytocin and vitamin D). Also antidepressant therapy, although prescribed only to few subjects of the total sample (N = 9), may have influenced the results of the present study. Other limitations are the cross-sectional design of the study, which does not allow to have precise information about the duration of both depression and obesity, the reduced sample size, and the collection of data in a single center.

Finally, this is an exploratory pilot study and further investigation is needed to confirm our results.

## Figures and Tables

**Table 1:** Characteristics of the study participants (N = 200).

Characteristics		Mean (±SD)
Age, years		52.7 (±12.9)
BMI, kg/m^2^		(33.8 (±5.5)
Homa-IR index		3.3 (±2.2)
Homocysteine, μmol/L		10.6 (±3.9)
% of neutrophils		59.3 (±7.7)
Oxytocin plasma levels, pg/mL		58.3 (39.1%)
BD-II score		15.9 (±10.1)
Vitamin D deficiency, n (%)	Yes	124 (62%)
	No	52 (26%)
	Not evaluable	24 (12%)
Severity of depression according to BD-II scores, n (%)	BD-II score <14-no depression	100 (50%)
	BD-II score 14–19-mild depression	13 (6.5%)
	BD-II score 20–29-moderate depression	74 (37%)
	BD-II score 30–63-severe depression	13 (6.5%)
Use of antidepressant, n (%)	Never	174 (87%)
	Currently	9 (4.5%)
	Previously	17 (8.5%)

BD-II: Beck Depression Inventory; BMI: Body Mass Index; HOMA-IR index: Homeostatic Model Assessment for Insulin Resistance.

**Table 2:** Association of BMI with levels of inflammatory parameters. In bold statistically significant *p*-values and q-FDR (False Discovery Rate).

Cytokine	Β ^1^	Lower CI	Upper CI	*p*-Value	q-FDR
GM_CSF	0.101	−0.014	0.216	0.086	0.201
IFN-γ	0.235	0.076	0.395	**0.004**	**0.056**
IL-2	0.025	−0.042	0.093	0.464	0.65
IL-3	3.70 × 10^−6^	−3.10 × 10^−6^	1.10 × 10^−5^	0.282	0.564
IL-4	0.004	−0.103	0.11	0.944	0.944
IL-6	0.043	0.009	0.076	**0.013**	**0.061**
IL-7	0.164	0.021	0.307	**0.024**	**0.084**
IL-8	0.218	−0.267	0.704	0.377	0.65
IL-10	0.016	−0.053	0.085	0.649	0.757
IL-18	1.307	−3.198	5.812	0.568	0.723
CCL2	0.415	−2.2	3.029	0.755	0.813
CCL3	1.613	0.342	2.884	**0.013**	**0.061**
TNF-α	0.148	0.01	0.286	**0.036**	**0.1**
TNF-β	0.022	−0.034	0.079	0.441	0.647

^1^ Models were adjusted for HOMA-IR index. Statistically significant values are reported in bold.

**Table 3:** Association of BMI with methylation of clock genes.

Log (Clock Genes)	β ^1^	Lower CI	Upper CI	*p*-Value	q-FDR
BMAL1	−0.002	−0.012	0.008	0.698	0.698
CLOCK	−0.005	−0.024	0.015	0.642	0.351
CRY1	0.01	−0.007	0.026	0.251	0.191
CRY2	0.012	−0.001	0.025	0.065	0.191
PER1	0.012	0	0.024	0.058	0.282
PER2	0.001	0	0.003	0.161	0.191
PER3	0.001	0	0.003	0.082	0.698

^1^ Multivariable regression models applied to logarithmic transformation of rate of methylation of clock genes, adjusted for HOMA-IR index, percentage of neutrophils, homocysteine plasma level, CpG dinucleotide position. BMAL1: Brain and Muscle Aryl Hydrocarbon Receptor Nuclear Translocator-like Protein-1; CI: Confidence Interval; CLOCK: Circadian Locomotor Output Cycles Kaput; CRY: Cryptochrome Circadian Regulator; PER: Period Circadian Regulator; q-FDR: False Discovery Rate.

## Data Availability

The data presented in this study is available upon request to the corresponding author.
